# The *Ralstonia solanacearum* Effector RipP1 Interacts with *Nicotiana benthamiana* FRL4a to Suppress Ethylene Signaling and Modulate Bacterial Wilt Susceptibility

**DOI:** 10.3390/plants15071039

**Published:** 2026-03-27

**Authors:** Xiaoyan Xie, Xue Ma, Jianwei He, Wenxia Hei, Baoling Zhang, Wenqi Huang, Xiaojing Fan, Mingfa Lv, Xiaofeng Zhang, Tao Zhuo

**Affiliations:** 1 College of Plant Protection, Fujian Agriculture and Forestry University, Fuzhou 350002, China; 2 State Key Laboratory of Agricultural and Forestry Biosecurity, Fujian Agriculture and Forestry University, Fuzhou 350002, China

**Keywords:** *Ralstonia solanacearum*, effector RipP1, FRL4a, HR, resistance to bacterial wilt

## Abstract

RipP1 is a well-characterized avirulence effector that induces a hypersensitive response (HR) in three tobacco species. However, the molecular mechanisms by which host proteins recognize RipP1 to activate a defense response and modulate host–pathogen interactions remain largely unknown. In this study, we screened a *Nicotiana benthamiana* cDNA library via yeast two-hybrid assay and identified FRIGIDA-like protein 4a (FRL4a) as a host protein interacting with RipP1. Secondary structure analysis of FRL4a and construction of serial mutants revealed that the ClyA-like domain of FRL4a is the key region mediating its interaction with RipP1. Using virus-induced gene silencing (VIGS) and quantitative real-time PCR (qPCR) analysis, we found that the ability of RipP1 to induce HR was significantly attenuated in FRL4a-silenced plants, and RipP1 no longer suppressed the ethylene signaling pathway. Pathogenicity tests by inoculating *R. solanacearum* on *N. benthamiana* with different *FRL4a* expression levels showed enhanced bacterial wilt resistance in *FRL4a*-silenced plants but increased susceptibility in *FRL4a*-overexpressing plants. Collectively, these findings demonstrate that RipP1 suppresses the ethylene pathway through its interaction with FRL4a, and FRL4a acts as a negative regulator of tobacco resistance to bacterial wilt.

## 1. Introduction

Bacterial wilt caused by *Ralstonia solanacearum* (currently reclassified as *Ralstonia pseudosolanacearum* in some phylogenetic lineages) is one of the most devastating soil-borne bacterial diseases worldwide, with a broad geographical distribution across tropical, subtropical, and temperate regions [1,2]. Ranked among the top ten most destructive bacterial plant pathogens worldwide, *R. solanacearum* infects over 310 plant species from 42 families, including economically important crops such as tobacco (*Nicotiana* spp.), potato (*Solanum tuberosum*), tomato (*S. lycopersicum*), and pepper (*Capsicum annuum*) [3,4]. Its wide host range and high pathogenicity pose significant challenges to agricultural production and cause substantial economic losses globally [5,6].

A hallmark of *R. solanacearum* pathogenicity is its dependence on the type III secretion system (T3SS), which delivers type III effector proteins (T3Es) into host plant cells [7,8,9]. To counteract microbial invasion, plants have evolved a multilayered immune system comprising pattern-triggered immunity (PTI) and effector-triggered immunity (ETI) [10,11]. However, successful pathogens deploy T3Es to suppress or evade these immune responses through diverse biochemical strategies, thereby promoting host colonization and disease development [12,13].

Over 100 T3Es have been identified in *R. solanacearum*, with individual strains carrying 60–75 effectors, representing one of the largest effector repertoires among known Gram-negative plant pathogenic bacteria [14,15,16]. Despite this extensive repertoire, the molecular functions of fewer than one-third of these effectors have been characterized, and only a limited number are known to activate ETI via well-defined recognition mechanisms [13,17].

To date, RipP2 (also known as PopP2) remains the only *R. solanacearum* effector demonstrated to directly interact with a plant resistance protein. RipP2 acetylates the RRS1 in *Arabidopsis thaliana*, thereby disrupting the paired TNL (Toll/interleukin-1 nucleotide-binding and leucine-rich repeat receptor) complex formed by RRS1 and RPS4 and activating RPS4-dependent immunity [18,19,20]. Other effectors, including RipE1, RipBN, RipP1 (also known as PopP1), and RipY, are recognized by distinct CC-NLR immune receptors in *N. benthamiana*: both RipE1 and RipBN are perceived by Ptr1 [21,22], whereas RipP1 and RipY are recognized by ZAR1 and RRS-Y, respectively [23,24]. RipBN is also recognized by Ptr1 in tomato (*S*. *lycopersicum*) and potato (*S*. *tuberosum*) [25,26]. Additionally, RipB is detected by the TIR-NLR protein Roq1 in *N. benthamiana* [27]. Notably, none of these effectors are directly recognized by their cognate R proteins through physical interaction.

RipP1 is a well-characterized avirulence effector that, together with RipAA, is recognized in at least three tobacco species, leading to HR and restriction of infection by *R. solanacearum* strain GMI1000 [28]. RipP1 in strain GMI1000 also serves as the avirulence determinant for *Petunia* St40 [29,30]. As a member of the YopJ/AvrRxv family of cysteine protease effectors, RipP1 requires an intact catalytic cysteine residue for HR induction [24,31], yet the molecular mechanism underlying its recognition in solanaceous plants remains poorly understood. Thus far, only the conserved CC-NLR protein NbZAR1 has been reported to participate in the indirect recognition of RipP1.

*N. benthamiana* FRIGIDA-like protein 4a (FRL4a) belongs to the FRIGIDA-LIKE (FRI) family, whose reported members are all involved in regulating plant flowering [32,33]. To date, no members of the FRI family have been reported to interact with phytopathogenic bacteria or to participate in regulating plant resistance to bacterial pathogens. Here, we identify FRL4a as a direct interacting partner of RipP1 in *N. benthamiana*. We demonstrate that FRL4a is required for full RipP1-induced HR and mediates RipP1-dependent suppression of ethylene (ET) signaling. Furthermore, inoculation assays with *R. solanacearum* on plants with varying *FRL4a* expression levels reveal that FRL4a negatively regulates plant resistance to *R. solanacearum*. Together, our findings uncover a previously unrecognized function of FRL4a in plant–bacterial interactions and provide mechanistic insight into RipP1-triggered immune responses in solanaceous plants.

## 2. Results

### 2.1. RipP1 Physically Interacts with FRL4a

**Figure 1:**
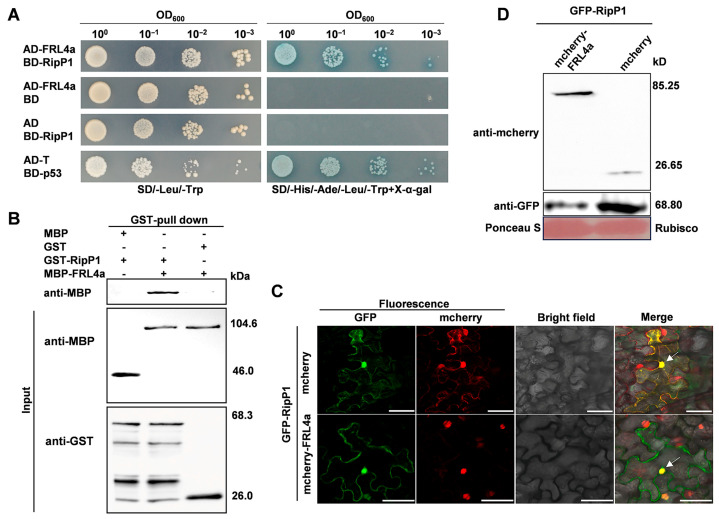
RipP1 interacts with *N. benthamiana* FRL4a in vivo and in vitro. (**A**) Y2H assay demonstrated the interaction between RipP1 and FRL4a. Transformants were adjusted to a cell density of OD_600_ = 1.0 and subjected to 10-fold serial dilution. For each dilution, 2 μL of transformants were spotted and incubated on SD/-Leu/-Trp plates for 2 d at 30 °C, and on SD/-His/-Ade/-Leu/-Trp plates supplemented with 20 μg/mL X-α-gal for 4 d at 30 °C. (**B**) In vitro interaction between RipP1 and FRL4a was verified by GST pull-down assay. Recombinant GST-RipP1 and MBP-FRL4a were subjected to GST pull-down analysis, and interacting proteins were detected by Western blotting. The experiment was repeated three times with consistent results. Negative controls included GST-RipP1 with MBP and GST with MBP-FRL4a. (**C**) RipP1 and FRL4a were co-localized in the nucleus. *N. benthamiana* leaves were agroinfiltrated for co-expression of GFP-fused RipP1 with mCherry-fused FRL4a or free mCherry. Green (GFP) and red (mCherry) fluorescence signals were captured at 36 hpi with excitation at 514 nm and 580 nm, respectively; merged green and red signals appear yellow. Bright-field images were acquired concurrently. Scale bars = 50 μm for all images. (**D**) Western blot validated the expression of the proteins using anti-GFP and anti-mCherry polyclonal antisera. Total proteins were extracted from agroinfiltrated leaf regions and separated by SDS-PAGE. Free mCherry was expressed from an empty vector as a control. Rubisco, stained with Ponceau S, served as the loading control.

Given that RipP1 functions as an avirulence effector and induces HR in tobacco, we hypothesized that RipP1 may be recognized through interaction with a host protein. To identify potential host targets, we conducted a yeast two-hybrid screen using RipP1 as the bait and a pool of *N. benthamiana* prey libraries. We aligned the DNA sequences with those from the *N. benthamiana* genome available in the database (https://solgenomics.net/organism/Nicotiana_benthamiana/genome (accessed on 21 March 2026)) and identified one candidate clone, *FRL4a* (*Niben101Scf07419g00003.1*), which encodes the protein FRIGIDA-Like 4a. To establish the relationship between the candidate gene and *ripP1*, the candidate clone and *ripP1* were constructed in the pGADT7 and pGBKT7 plasmids, respectively, and subsequently retransformed into yeast. Yeast containing pGADT7-FRL4a and pGBKT7-RipP1 exhibited normal growth on SD medium lacking His, Ade, Leu, and Trp, supplemented with X-α-galactosidase (SD/-His/-Ade/-Leu/-Trp+X-α-gal), as well as on SD medium deficient in Leu and Trp (SD/-Leu/-Trp), closely resembling the positive control (pGADT7-T + pGBKT7-p53) (Figure 1A). This result indicates that RipP1 interacts with FRL4a in yeast. To further validate the interaction observed in yeast, GST pull-down assays were performed. In the control lanes, GST protein did not pull down MBP-FRL4a protein or GST-RipP1 protein did not pull down MBP protein, whereas GST-RipP1 protein successfully pulled down MBP-FRL4a protein (Figure 1B).

To further elucidate the relationship between RipP1 and FRL4a, we conducted studies to investigate the subcellular co-localization of these proteins in *N. benthamiana* leaves. GFP-tagged RipP1 and mCherry-tagged FRL4a were co-expressed in *N. benthamiana* leaves. Confocal microscopy analysis revealed that the fluorescence of the two proteins was merged and exhibited clear nuclear localization (Figure 1C). Western blotting results confirmed the correct expression of both proteins in the inoculated leaves (Figure 1D). The co-localization of RipP1 and FRL4a provides supporting evidence for their interaction within the tobacco nucleus.

### 2.2. The ClyA-like Superfamily Domain of FRL4a Mediates Interaction with RipP1

**Figure 2:**
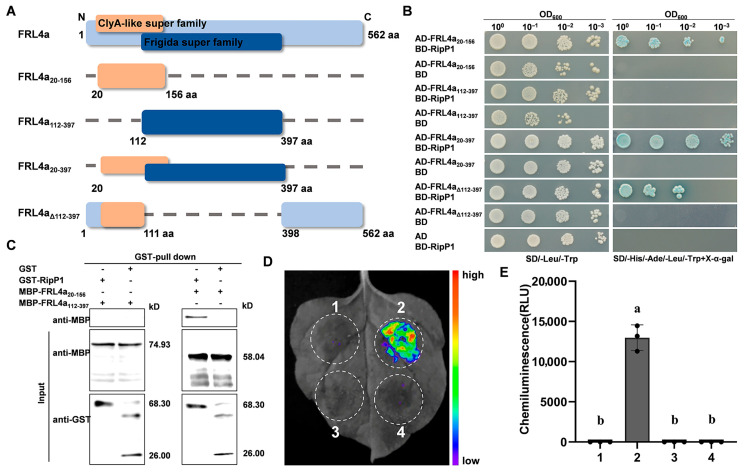
The ClyA-like superfamily domain of FRL4a is essential for its interaction with RipP1. (**A**) Schematic diagram of the domain architecture of FRL4a and its truncated derivatives (FRL4a_20–156_, FRL4a_112–397_, FRL4a_20–397_) and deletion mutant (FRL4aΔ_112–397_). Amino acid residue positions are indicated by numbers. The ClyA-like superfamily domain is shown as an orange box, and the FRIGIDA superfamily domain as a dark blue box. Deleted amino acid regions in FRL4a are marked with a dashed line. (**B**) Y2H assay confirmed that FRL4a_20–156_ was required for the FRL4a-RipP1 interaction. Transformant preparation, serial dilution, spotting and incubation conditions were identical to those described in Figure 1A. (**C**) GST pull-down assay verified the interaction between FRL4a_20–156_ and RipP1. Recombinant GST-RipP1, MBP-FRL4a_20–156_ and MBP-FRL4a_112–397_ were used for GST pull-down analysis, with interacting proteins detected by Western blotting. The experiment was repeated three times with consistent results. GST protein with MBP-FRL4a_20–156_ or MBP-FRL4a_112–397_ served as negative controls. (**D**) Split luciferase assay confirmed the interaction between FRL4a_20–156_ and RipP1. Chemiluminescence images were captured at 48 hpi following application of 0.5 μM luciferin. Three biological replicates yielded similar results. *N. benthamiana* leaf regions co-infiltrated with *Agrobacterium* carrying 35S:FRL4a_20-156_-nLUC and 35S:cLUC-GFP, 35S:FRL4a_20-156_-nLUC and 35S:cLUC-RipP1_C229A_, 35S:FRL4a_112-397_-nLUC and 35S:cLUC-GFP, and 35S:FRL4a_112-397_-nLUC and 35S:cLUC-RipP1_C229A_ are labeled 1 to 4, respectively. (**E**) Quantification of total photon counts over 5 min for the samples in (**D**), the letters (a,b) are used to indicate significant differences in the ANOVA analysis,. Values are presented as mean ± SD (*n* = 3 biological replicates; one-way ANOVA with Tukey’s test, *p* < 0.01).

The *FRL4a* gene is predicted to encode a polypeptide consisting of 562 residues, which contains both a ClyA-like superfamily domain and a Frigida superfamily domain at the N-terminus (Figure 2A). To determine which region of FRL4a mediates interaction with RipP1, a series of truncated FRL4a constructs was generated (Figure 2A) and tested in Y2H assays. All truncated FRL4a clones were individually inserted into the pGADT7 plasmid and subsequently transformed into yeast containing the pGBKT7-RipP1 plasmid. While all yeast strains grew normally on SD/-Leu/-Trp, only those harboring pGADT7-FRL4a_20–156_ and pGBKT7-RipP1, pGADT7-FRL4a_20-397_ and pGBKT7-RipP1, or pGADT7-FRL4a_Δ112-397_ and pGBKT7-RipP1 were able to grow on SD/-His/-Ade/-Leu/-Trp + X-α-gal (Figure 2B). Moreover, yeast containing pGADT7-FRL4a_Δ112-397_ which encoded a C-terminal truncated ClyA-like super family domain and pGBKT7-RipP1 exhibited a marked growth attenuation compared to those with pGADT7-FRL4a_20-156_ and pGBKT7-RipP1, or pGADT7-FRL4a_20-397_ and pGBKT7-RipP1 on SD/-His/-Ade/-Leu/-Trp + X-α-gal (Figure 2B). These results indicate that the ClyA-like superfamily domain is required for the interaction between FRL4a and RipP1.

To confirm the interaction between the ClyA-like superfamily domain of FRL4a and RipP1 observed in yeast, we conducted GST pull-down assays. The GST-RipP1 protein did not pull down the MBP-FRL4a_112-397_ protein; however, it successfully pulled down the MBP-FRL4a_20-156_ protein (Figure 2C). Additionally, we utilized split luciferase assays to provide further evidence for the in vivo interaction between FRL4a_20-156_ and RipP1. The expression of RipP1 in tobacco leaves induced a strong HR, prompting the identification of a mutant RipP1_C229A_ that abolished RipP1-mediated cell death while maintaining its binding to FRL4a (Figure S1A,B). Split luciferase assays demonstrated intense luminescence in leaf regions co-expressing RipP1_C229A_ and FRL4a_20-156_, whereas the co-expression of RipP1_C229A_ with FRL4a_112-397_ or negative controls resulted in negligible luminescence (Figure 2D,E). Together, these data indicate that the ClyA-like superfamily domain of FRL4a is necessary and sufficient for interaction with RipP1.

### 2.3. FRL4a Contributes to RipP1-Induced Hypersensitive Response

To determine whether FRL4a is required for RipP1-induced HR, a Tobacco Rattle Virus (TRV)-based virus-induced gene silencing (VIGS) system was employed [34]. To suppress the function of FRL4a using TRV-VIGS, a 348 bp fragment of the *FRL4a* gene was cloned into TRV-RNA2 (pTRV2). A mixture of *Agrobacterium* GV3101 cultures containing TRV-RNA1 (pTRV1) and pTRV2 with the *FRL4a* T-DNA (pTRV2:*FRL4a*) constructs was infiltrated into the leaves of 3-week-old *N. benthamiana*. For the negative control, a mixture of *Agrobacterium* GV3101 cultures containing TRV-RNA1 (pTRV1) and pTRV2 (pTRV2:*gfp*), which carried the *gfp* gene T-DNA construct, was used. Ten days post-Agro-infiltration, three concentrations (OD_600_ = 0.3, 0.03, and 0.003) of the transformed GV3101 strain harboring the pGDGm:RipP1 plasmid, which expresses RipP1 with a C-terminal GFP fusion, were infiltrated onto the upper leaves of TRV-infected plants. In parallel, we confirmed that C-terminal GFP fusion to RipP1 (RipP1-GFP) did not impair its ability to induce HR in *N. benthamiana* (Figure S2).

**Figure 3:**
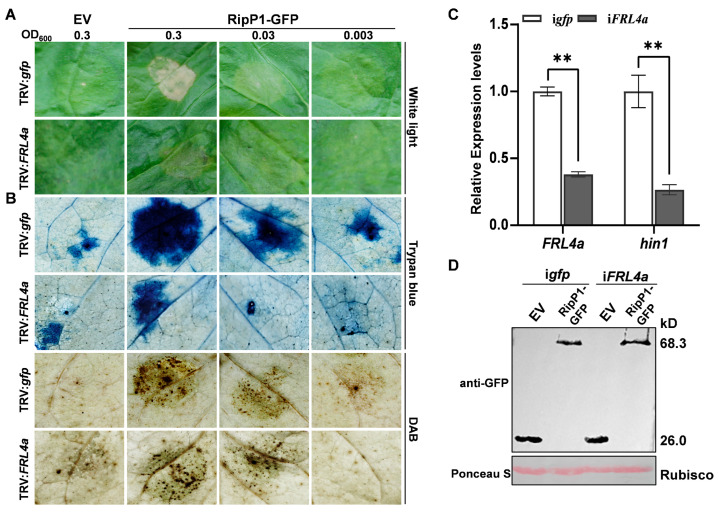
TRV-mediated *FRL4a* silencing attenuates the necrosis phenotype in tobacco plants. (**A**) Transient expression of RipP1-GFP in *FRL4a*-silenced *N. benthamiana* leaves. *A*. *tumefaciens* GV3101 harboring RipP1 was adjusted to OD_600_ = 0.3, 0.03 and 0.003; GV3101 harboring the empty vector pGDGm was adjusted to OD_600_ = 0.3. These cultures were agroinfiltrated into *FRL4a*-silenced and *gfp*-silenced *N. benthamiana* plants (negative control). Photographs were taken at 2 dpi. (**B**) Detection of plant cell death and ROS accumulation. Tobacco leaves were stained with trypan blue (cell death) and DAB (ROS) at 20 hpi. (**C**) qPCR analysis showed the transcript levels of *FRL4a* and *hin1* in TRV-mediated *FRL4a*-silenced *N. benthamiana* plants. Leaves were agroinfiltrated with GV3101 harboring pGDGm (empty vector) or pGDGm:RipP1 at OD_600_ = 0.03 for 2 d. Light columns: control plants (i*gfp*); dark columns: *FRL4a*-silenced plants (i*FRL4a*). Error bars represent SD from three independent experiments. Statistical significance was determined by Student’s *t*-test (** *p* < 0.001). (**D**) Western blot analysis using anti-GFP polyclonal antiserum. Total proteins were extracted from agroinfiltrated regions of control and i*FRL4a* plants expressing free GFP (empty vector) or RipP1-GFP (pGDGm:RipP1), and separated by SDS-PAGE. Rubisco, stained with Ponceau S, served as the loading control.

After two days, the leaves of TRV:*FRL4a* plants agroinfiltrated for expression of RipP1-GFP exhibited significantly weaker necrotic phenotypes compared to those of TRV:*gfp* plants agroinfiltrated under the same conditions (Figure 3A). Trypan blue and DAB staining at 20 hpi revealed that *FRL4a*-silenced leaves agroinfiltrated with RipP1-GFP-expressing *Agrobacterium* displayed fainter blue staining and significantly less hydrogen peroxide accumulation than control leaves expressing RipP1-GFP (Figure 3B). qPCR was conducted to confirm the silencing of *FRL4a* in the infiltrated leaves. The qPCR results indicated that the expression level of *FRL4a* in the infiltrated leaves of TRV:*FRL4a*-infected plants was considerably lower than that in the infiltrated leaves of TRV:*gfp* (Figure 3C), confirming successful silencing of *FRL4a* by TRV-VIGS. Concurrently, qPCR analysis of the HR marker gene *hin1* in leaves inoculated with the transformed GV3101 strain harboring the pGDGm:RipP1 plasmid at 48 hpi demonstrated that *hin1* expression in *FRL4a*-silenced leaves was reduced by approximately 73% compared to control leaves (Figure 3C). Furthermore, Western blot analysis confirmed comparable accumulation of RipP1 protein in both *FRL4a*-silenced and control plants (Figure 3D). These results demonstrate that FRL4a is required for full RipP1-induced HR in *N. benthamiana*.

### 2.4. RipP1 Suppresses Ethylene Signaling Through FRL4a

The plant HR is closely linked to the initiation of defense-related mechanisms, wherein hormone signaling pathways mediated by salicylic acid (SA), jasmonic acid (JA), and ET play essential regulatory roles [35,36,37]. Since RipP1 induces a strong HR in *N. benthamiana* leaves, we performed qPCR to quantify the expression levels of SA marker genes (*PR1* and *PR2*), JA marker genes (*Coi1* and *MYC2*), and ET marker genes (*EIN2* and *EIN3*) in *N. benthamiana* leaves agroinfiltrated with pGDGm:RipP1, using leaves agroinfiltrated with the empty vector pGDGm as a control. The results showed that the transcript levels of *PR1* and *PR2* were significantly elevated in leaves expressing RipP1 compared with the control, whereas the expression levels of *Coi1*, *MYC2*, *EIN2*, and *EIN3* were markedly reduced (Figure S3). These results indicate that RipP1 induces the SA signaling pathway while suppressing the JA and ET signaling pathways in *N. benthamiana*.

**Figure 4:**
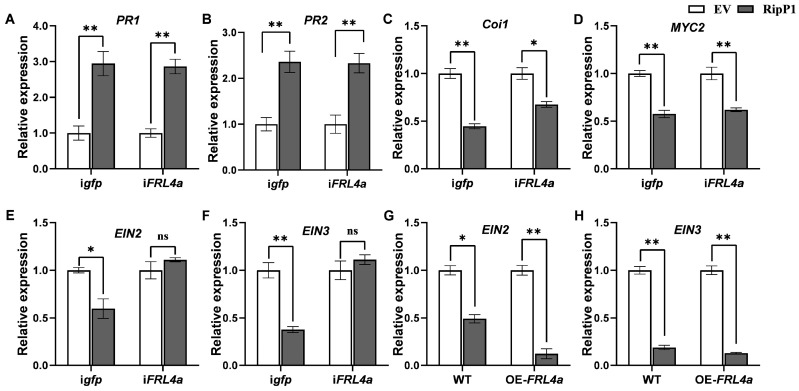
Silencing of *FRL4a* impairs the inhibition of ET signaling response by RipP1 in *N. benthamiana.* (**A**,**B**) RipP1 induces the expression of *PR1* (**A**) and *PR2* (**B**) in both control and i*FRL4a* plants. (**C**,**D**) RipP1 represses the expression of *COi1* (**C**) and *MYC2* (**D**) in both control and *iFRL4a* plants. (**E**,**F**) FRL4a modulates RipP1-mediated suppression of *EIN2* (**E**) and *EIN3* (**F**) in *N. benthamiana*. (**G**,**H**) RipP1 inhibits *EIN2* (**G**) and *EIN3* (**H**) in both wild-type (WT) and FRL4a-overexpressing (OE-FRL4a) transgenic *N. benthamiana* plants. Light columns: Leaves were agroinfiltrated with GV3101 harboring pGDGm (empty vector); dark columns: Leaves were agroinfiltrated with GV3101 harboring pGDGm:RipP1. *N. benthamiana* leaves were infiltrated with the above strains at an OD_600_ of 0.03. Total RNA was isolated at 24 hpi. Error bars represent SD from three independent experiments. Statistical significance was determined by Student’s *t*-test (** *p* < 0.001, * *p* < 0.05, ns = not significant, *p* ≥ 0.05).

Given that RipP1 interacts with FRL4a and induces a weaker HR in *FRL4a*-silenced plants, we investigated whether FRL4a is involved in the RipP1-mediated modulation of SA, JA, or ET pathways. To this end, we assessed whether RipP1 retained its ability to induce the SA pathway and repress the JA and ET pathways in *FRL4a*-silenced leaves. Leaves of *FRL4a*-silenced plants, in which the efficiency of silencing was confirmed by qPCR, along with control plants (TRV:*gfp* plants) (Figure S4), were agroinfiltrated with pGDGm:RipP1. Leaves agroinfiltrated with the empty vector pGDGm served as controls. The results indicated that, in both control and *FRL4a*-silenced plants, the expression of *PR1* and *PR2* in RipP1-expressing leaves was significantly upregulated compared to that in empty vector-infiltrated leaves (Figure 4A,B). Likewise, RipP1 suppressed the expression of *Coi1* and *MYC2* in both control and *FRL4a*-silenced plants (Figure 4C,D). These findings demonstrate that RipP1 expression consistently induced transcriptional changes in wild-type (WT) plants, as well as in both control and *FRL4a*-silenced plants, compared with the empty vector, suggesting that *FRL4a* silencing did not influence RipP1-mediated induction of the SA signaling pathway or suppression of the JA signaling pathway in tobacco.

In line with the results in WT plants, RipP1 expression markedly decreased the transcript levels of *EIN2* and *EIN3* in control (TRV:*gfp*) plants compared to empty vector-agroinfiltrated leaves (Figure 4E,F). However, in *FRL4a*-silenced plants, the expression levels of *EIN2* and *EIN3* in RipP1-expressing leaves showed no significant difference compared to those in empty vector-agroinfiltrated leaves (Figure 4E,F). Consistent results were obtained using *FRL4a*-overexpressing transgenic lines, in which RipP1-induced suppression of *EIN2* and *EIN3* was further intensified (Figure 4G,H). The expression level of *FRL4a* in transgenic plants was validated through qPCR (Figure S4). Collectively, these findings suggest that silencing of *FRL4a* disrupts RipP1-mediated suppression of ET response in tobacco, thereby indicating that FRL4a is specifically essential for RipP1-mediated suppression of the ET signaling pathway.

### 2.5. FRL4a Negatively Regulates Resistance to Bacterial Wilt

**Figure 5:**
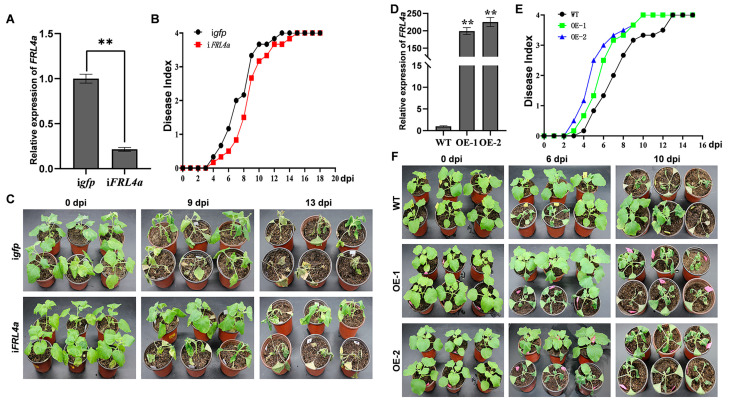
FRL4a enhances the susceptibility of *N. benthamiana* plants to *Ralstonia solanacearum* strain FJ1003. (**A**) qPCR analysis showed *FRL4a* transcript levels in TRV-mediated i*FRL4a* plants. i*gfp*: plants infiltrated with a mixture of *Agrobacterium* GV3101 cultures containing pTRV1 and pTRV2:*gfp*; *iFRL4a*: plants infiltrated with a mixture of *Agrobacterium* GV3101 cultures containing pTRV1 and pTRV2:*FRL4a*. Error bars represent SD from three independent experiments. Differences were evaluated using Student’s *t*-tests (** *p*< 0.001). (**B**) Progression of bacterial wilt in *R. solanacearum* FJ1003-inoculated tobacco plants. Wilt symptom severity was scored daily from 0 to 18 dpi using a disease index after root inoculation. Each data point represents the mean disease index of six plants per treatment. (**C**) Bacterial wilt symptoms in plants from (**B**). Photographs were taken at 0, 9 and 13 dpi. All experiments were repeated three times with consistent results; representative images are shown. (**D**) qPCR analysis showed *FRL4a* transcript levels in *FRL4a*-overexpressing transgenic *N. benthamiana* plants. WT: wild-type plants; OE-1 and OE-2: two independent FRL4a-overexpressing transgenic lines. Error bars represent SD from three independent experiments. Differences were evaluated using Student’s *t*-tests (** *p* < 0.001). (**E**) Progression of bacterial wilt in FJ1003-inoculated WT and OE-FRL4a plants. Wilt symptom severity was scored daily from 0 to 15 dpi using a disease index after root inoculation. Each data point represents the mean disease index of six plants per treatment. (**F**) Bacterial wilt symptoms in plants from (**E**). Photographs were taken at 0, 6 and 10 dpi. All experiments were repeated three times with consistent results; representative images are shown.

To elucidate the role of FRL4a in the resistance of *N. benthamiana* against *R. solanacearum* infection, we assessed the susceptibility of *FRL4a*-silenced plants, in which the efficiency of silencing was confirmed by qPCR (Figure 5A), alongside control plants (TRV:*gfp* plants) to bacterial wilt. Tobacco roots were inoculated with the *R. solanacearum* FJ1003 strain via soil drenching. At 4 dpi, both *FRL4a*-silenced and control plants exhibited wilting, with average disease indices of approximately 0.17 and 0.33, respectively (Figure 5B). Subsequent disease progression indicated consistently lower severity in *FRL4a*-silenced plants, particularly between 6 and 9 dpi, during which these plants demonstrated a significantly lower disease index compared to control plants (Figure 5B,C). By 13 dpi, control plants had completely collapsed, reaching the maximum disease index score of 4, whereas *FRL4a*-silenced plants exhibited an average index of 3.83, achieving full wilting only by 16 dpi (Figure 5B,C). These results demonstrate that silencing *FRL4a* enhances resistance to *R. solanacearum* in *N. benthamiana*.

*FRL4a*-overexpressing transgenic plants were evaluated for their resistance to bacterial wilt. qPCR analysis revealed significant increases in *FRL4a* transcript levels in both transgenic lines compared to wild-type plants (Figure 5D). No obvious morphological differences were observed between the *FRL4a*-overexpressing transgenic lines and wild-type plants under normal growth conditions, including seed germination rate, germination timing, plant height, and leaf shape. When these plants were inoculated with *R. solanacearum* FJ1003, the transgenic lines exhibited accelerated disease development, with symptom onset occurring at 3 dpi compared to 4 dpi in wild-type plants (Figure 5E). Initial disease indices were recorded at 1.17 for OE-1 and 0.67 for OE-2, while wild-type plants had a disease index of only 0.17 (Figure 5E). The enhanced susceptibility phenotype was particularly pronounced between 5 and 8 dpi, with both overexpression lines demonstrating significantly increased disease progression (Figure 5E,F). At 10 dpi, both transgenic lines reached complete wilting (disease index: 4), whereas wild-type plants averaged a disease index of 3.33, achieving full wilting only at 13 dpi (Figure 5E,F). The disease indices of both transgenic lines were higher than those of wild-type plants, indicating that FRL4a overexpression enhances susceptibility to *R. solanacearum* in *N. benthamiana*. These findings illustrate that FRL4a acts as a negative regulator of defense against bacterial wilt.

### 2.6. FRL4a Is Conserved in Solanaceae and Predominantly Expressed in Leaves

**Figure 6:**
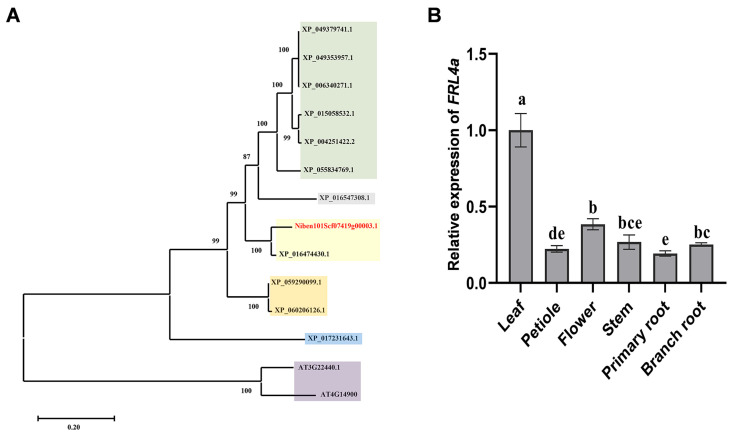
Phylogenetic analysis of FRL4a and its tissue-specific expression pattern in *N. benthamiana.* (**A**) Maximum likelihood phylogenetic tree of NbFRL4a and its homologous proteins from *Solanum stenotomum* (XP_049379741.1), *Solanum verrucosum* (XP_049353957.1), *Solanum tuberosum* (XP_006340271.1), *Solanum pennellii* (XP_015058532.1), *Solanum lycopersicum* (XP_004251422.2), *Solanum dulcamara* (XP_055834769.1), *Capsicum annuum* (XP_016547308.1), *Nicotiana tabacum* (XP_016474430.1), *Lycium ferocissimum* (XP_059290099.1), *Lycium barbarum* (XP_060206126.1), *Daucus carota* subsp. *sativus* (XP_017231643.1) and *Arabidopsis thaliana* (AT3G22440.1, AT4G14900). Bootstrap values (percentage of 1000 replicates) are shown at branch nodes. The scale bar indicates 0.2 amino acid substitutions per site. FRL4a homologs from the same genus are highlighted with identical background colors; NbFRL4a is labeled in red font. (**B**) qPCR analysis of *FRL4a* transcript levels in different Nb tissues. The letters (a–e) are used to indicate significant differences in the ANOVA analysis. Values are presented as mean ± SD (*n* = 3 biological replicates; one-way ANOVA with Tukey’s test, *p* < 0.01).

To determine the conservation of FRL4a among Solanaceae species, a BLAST (https://blast.ncbi.nlm.nih.gov/Blast.cgi (accessed on 21 March 2026)) search was conducted using the *N. benthamiana* FRL4a amino acid sequence as a query in the NCBI (https://www.ncbi.nlm.nih.gov/nucleotide/ (accessed on 21 March 2026)) and *Arabidopsis* databases (http://www.arabidopsis.org/ (accessed on 21 March 2026)) to identify homologous proteins. A phylogenetic tree was subsequently constructed for cluster analysis. The phylogenetic analysis indicated that FRL4a is widely distributed across Solanaceae species, with greater sequence similarity observed among closely related species (Figure 6A), suggesting stronger conservation. The highest homology of FRL4a was noted between *N. benthamiana* and *N*. *tabacum* (92.72% identity), while the lowest homology was observed between *N. benthamiana* and *A*. *thaliana* (28.10% identity). Notably, FRL4a sequences demonstrated 84.57% identity within Solanaceae species, with particularly high conservation (89.02% identity) in the Frigida superfamily domain (Figure S5). These findings indicate that FRL4a is both prevalent and evolutionarily conserved in Solanaceae plants.

The expression pattern of *FRL4a* in various *N. benthamiana* tissues was analyzed using qPCR. The results revealed that *FRL4a* expression was highest in leaves and lowest in primary roots, with leaf expression being approximately 5.17-fold higher than in primary roots and 2.60-fold higher than in flowers (Figure 6B). This expression pattern correlates with the mosaic and chlorosis phenotypes observed in *FRL4a*-silenced *N. benthamiana* leaves (Figure S6).

## 3. Discussion

*R. solanacearum* deploys a diverse repertoire of effector proteins to manipulate host cellular processes [15]. Notably, individual effectors frequently target multiple subcellular compartments, thereby exerting distinct biological functions through different mechanisms [4,17]. Such functional versatility likely reflects the ongoing co-evolutionary arms race between plants and pathogens. Previous studies have shown that RipP1 is indirectly recognized by the NLR protein NbZAR1 and pseudokinase NbJIM2 without detectable physical interaction, although both proteins modulate RipP1-induced HR [24]. In contrast, our study identifies FRL4a as a direct interacting partner of RipP1 and demonstrates its involvement in RipP1-elicited cell death in *N. benthamiana*. Importantly, mutation of the conserved catalytic cysteine residue in the cysteine protease domain of RipP1 abolishes its HR-inducing activity but does not disrupt its interaction with NbFRL4a. This dissociation between enzymatic activity and protein–protein interaction suggests that RipP1 recognition and HR activation likely involve additional host factors beyond FRL4a in *N. benthamiana*.

NbFRL4a belongs to the FRI family, members of which have been primarily implicated in the regulation of flowering time [32,33]. To our knowledge, this study provides the first evidence that an FRI family member directly recognizes a bacterial type III effector and participates in effector-triggered HR. In *A*. *thaliana*, the closest homologs of NbFRL4a are AtFRL4a and AtFRL4 (Figure 6A), which share sequence identities of 28.1% and 27.51%, respectively, and are paralogous genes exhibiting 80.56% sequence similarity. Overexpression of *AtFRL4* in *N*. *tabacum* delays flowering, and TaFRL4a, the wheat ortholog of AtFRL4a, similarly represses flowering [38,39]. By contrast, neither silencing nor overexpression of *NbFRL4a* in *N. benthamiana* resulted in detectable alterations in flowering time. This functional divergence may be attributable to the relatively low sequence identity between the FRIGIDA superfamily domain of NbFRL4a and those of AtFRL4a and AtFRL4 (32.77% and 32.31%, respectively). Consistent with this interpretation, phylogenetic analysis of FRL4a homologs across multiple plant species reveals a substantial genetic distance between *N. benthamiana* and *A*. *thaliana* FRL4a proteins (Figure 6A). Collectively, these results expand the functional landscape of FRIGIDA-LIKE proteins beyond flowering regulation.

Our data further demonstrate that NbFRL4a interacts with the *R. solanacearum* effector RipP1 via its conserved ClyA-like superfamily domain. This domain represents a structural motif characteristic of the α-pore-forming toxin (α-PFT) family [40,41]. In bacterial pathogens of animals, pore-forming toxins are well-established virulence factors that disrupt host cell membrane integrity [42]; however, their relevance in plant–pathogen interactions remains poorly understood [43,44]. Intriguingly, several plant species, including *Enterolobium contortisiliquum* and *Triticum aestivum*, have been reported to encode PFT-like proteins, although their biological roles in plants are largely unknown [45,46]. Notably, sequence analysis indicates that the ClyA-like domain is uniquely present in NbFRL4a and its tobacco ortholog NtFRL4a but absent from other Solanaceae FRL4a homologs. These observations raise the possibility that the ClyA-like domain confers a specialized function to FRL4a, potentially enabling direct recognition of RipP1. Further studies will be required to determine how this domain contributes to FRL4a function and to clarify its role in plant–pathogen interactions.

The JA, SA, and ET signaling pathways constitute central regulatory networks in plant defense against microbial pathogens [37]. Among them, SA signaling generally antagonizes JA and ET pathways and positively regulates resistance to biotrophic and hemibiotrophic pathogens, including *R. solanacearum* [47,48,49]. In agreement with this regulatory framework, our results show that RipP1 induces the expression of SA-responsive marker genes while simultaneously suppressing the transcription of JA- and ET-responsive genes. This pattern is consistent with previous reports on the *R. solanacearum* avirulence effectors RipAA from strain GMI1000 and RipB from strain RS1002, both of which also activate SA signaling [27,50].

Furthermore, our findings establish that FRL4a counteracts RipP1-mediated suppression of the ET pathway, and that this interference accounts for the attenuation of RipP1-induced HR observed in FRL4a-silenced leaves (Figure 4E,F). This finding highlights a critical role for ET signaling in RipP1-mediated ETI. Notably, earlier studies have shown that RipP1-induced HR is compromised in *Nbeds1* mutant plants, which are defective in SA-dependent immune signaling [24,51]. Taken together, these findings suggest that RipP1 activates HR through multiple, mechanistically distinct pathways involving coordinated modulation of SA- and ET-associated immune responses.

Although FRL4a is required for RipP1-induced HR, our results also indicate that FRL4a negatively regulates tobacco resistance to bacterial wilt. These seemingly paradoxical phenotypes imply that FRL4a mediates RipP1-triggered HR and represses bacterial wilt resistance through distinct biological functions that are likely governed by separate molecular mechanisms. As previously described, HR not only induces local cell death at the infection site but also triggers systemic defense signals, thereby promoting the development of systemic acquired resistance (SAR) and conferring overall disease resistance in plants [52]. Based on this, we hypothesize that FRL4a may modulate plant SAR through other interacting partners. To explore this possibility, we performed a yeast two-hybrid screen using FRL4a as bait against a *N. benthamiana* cDNA library, which identified P58^IPK^, a member of the DnaJ-like protein family, as an interacting partner of FRL4a (Figure S7). P58^IPK^ has been characterized as a positive regulator of antiviral immunity in both *A*. *thaliana* and *N. benthamiana*, and loss of P58^IPK^ function increases plant susceptibility to viral infection [53]. In contrast, its role in plant defense against bacterial pathogens remains unknown. Ongoing work in our laboratory is focused on determining whether FRL4a represses bacterial wilt resistance by interacting with P58^IPK^ and thereby interfering with its pro-defense activity. In addition, during infection, *R. solanacearum* delivers 60–75 effectors into host cells via the T3SS, not just RipP1 [15]. Other effectors may interfere with or suppress the RipP1–FRL4a module, which is also part of our future research.

In summary, our study reveals two distinct biological functions of FRL4a in *N. benthamiana*. First, FRL4a directly recognizes the *R. solanacearum* effector RipP1 in the plant cell nucleus via its ClyA-like superfamily domain. This interaction is required for RipP1-mediated suppression of the ET signaling pathway, which in turn enables the induction of a robust HR in plant leaves. Consistently, silencing of *FRL4a* abolishes RipP1-dependent ET suppression and concomitantly weakens HR. Second, FRL4a functions as a negative regulator of plant resistance to bacterial wilt, as *FRL4a*-silenced plants exhibit enhanced resistance to *R. solanacearum*, whereas *FRL4a*-overexpressing plants display increased susceptibility. Together, these findings uncover a previously unrecognized role for FRL4a that is distinct from those of characterized FRIGIDA-LIKE family members, define a new pathway underlying RipP1-induced HR, and provide insights that may facilitate the identification and deployment of bacterial wilt resistance genes in Solanaceae crops.

## 4. Materials and Methods

### 4.1. Plant and Bacterial Materials

*N. benthamiana* plants were cultivated in soil under controlled conditions of 24 ± 1 °C with a 16-h light/8-h dark photoperiod, where the light intensity was maintained at approximately 7.5 μmol·m^−2^·s^−1^. All bacterial strains used in this study are detailed in Table S1. *Ralstonia solanacearum* strain FJ1003 was cultured at 28 °C in Nutrient-rich broth (NB), whereas *Escherichia coli* DH5α and *Agrobacterium tumefaciens* GV3101 were grown in Luria broth (LB) medium at 37 °C and 28 °C, respectively.

### 4.2. Yeast Two-Hybrid Assay

A cDNA library of *N. benthamiana* was constructed into the pGADT7 (AD) vector provided by the manufacturer. RipP1 was cloned into the pGBKT7 (BD) vector at the *Nde*I/*Eco*RI restriction sites to generate BD-RipP1. The yeast two-hybrid assay was performed in accordance with the manufacturer’s instructions (Takara Bio, CA, USA), and positive transformants were screened on SD medium lacking His, Ade, Leu, and Trp (SD/-His/-Ade/-Leu/-Trp). To investigate the interaction between RipP1 and FRL4a or FRL4a’s truncation mutants, the coding sequences of FRL4a, FRL4a_20-156_, FRL4a_112-397_, FRL4a_20-397_, and FRL4a_Δ112-397_ were individually cloned into pGADT7. Subsequently, the plasmid combinations BD-RipP1/AD-FRL4a, BD-RipP1/ADFRL4a_20-156_, BD-RipP1/ADFRL4a_112-397_, BD-RipP1/ADFRL4a_20-397_, and BD-RipP1/ADFRL4a_Δ112-397_ were co-transformed into the yeast strain AH109. The transformants were cultured on SD medium lacking Leu and Trp (SD/-Leu/-Trp) at 30 °C for 2 days, or on SD/-His/-Ade/-Leu/-Trp plates at 30 °C for 4 days for screening. The interaction between RipP1 and FRL4a, FRL4a_20-156_, FRL4a_20-397_, or FRL4a_Δ112-397_ was confirmed by incubating the transformants on SD/-His/-Ade/-Leu/-Trp plates supplemented with 20 μg/mL X-α-galactosidase (X-α-gal). Cultured transformants were adjusted to a cell density of OD_600_ = 1.0, and then serially diluted 10-fold. For each dilution series, 2-μL aliquots of the cell suspensions were spotted onto the corresponding plates, which were then incubated at 30 °C for 4 days. Positive controls included the pGADT7-T and pGBKT7–53, while negative controls consisted of the empty pGBKT7 and pGADT7 vectors, as well as pGADT7 constructs expressing FRL4a and its truncation mutants.

### 4.3. GST Pull-Down Assay

The full-length *ripP1* fragment was excised from the BD-RipP1 plasmid by digestion with *Eco*RI and *Nde*I, and subsequently cloned into the *Eco*RI/*Nde*I-linearized pGEK-GX vector to generate the recombinant plasmid GST-RipP1. The coding sequences of FRL4a, FRL4a_20-156_, and FRL4a_112-397_ were amplified from their corresponding AD vectors and inserted between the *Eco*RI and *Bam*HI restriction sites of the pMAL-c2x vector, resulting in MBP-FRL4a, MBP-FRL4a_20-156_, and MBP-FRL4a_112-397_, respectively. For GST pull-down, GST- and MBP-tagged proteins were purified separately using glutathione agarose (GE Healthcare Bio-Sciences, Uppsala, Sweden) and amylose affinity chromatography (NEB, Ipswich, UK), respectively. GST-pull-down assays were then performed as described previously [54]. Briefly, 3 μg of GST-RipP1 was co-incubated overnight at 4 °C with 3 μg of MBP-FRL4a, MBP-FRL4a_20-156_ or MBP-FRL4a_112-397_, along with 50 μL of glutathione agarose. Proteins bound to the glutathione agarose were eluted, boiled in 2 × SDS loading buffer, and separated by a 12% sodium dodecylsulfate-polyacrylamide gel electrophoresis (SDS-PAGE). Subsequently, the separated proteins were transferred onto a NC membrane and immunoblotted with an anti-MBP antibody. Negative controls were included: 3 μg of GST protein was co-incubated with MBP-FRL4a, MBP-FRL4a_20-156_, or MBP-FRL4a_112-397_, and 3 μg of MBP protein was co-incubated with GST-RipP1, following the same incubation and detection procedures.

### 4.4. Split-Luciferase Complementation Assay

A split-luciferase complementation assay was performed to detect the in vivo interaction between RipP1_C1229A_ and FRL4a_20-156_, or between RipP1_C1229A_ and FRL4a_112-397_, as described previously [55]. The coding sequences of FRL4a_20-156_ and FRL4a_112-397_ were amplified from their corresponding AD vectors and individually fused with cLUC at the C terminus. Subsequently, FRL4a_20-156_ or FRL4a_112-397_ was co-expressed with nLUC-RipP1 in the leaves of four-week-old *N. benthamiana* plants. Leaves were collected at 40 hpi, treated with 0.5 mM luciferin, and dark-adapted for 1 min to quench fluorescence. Luciferase luminescence images were captured using a cooled CCD imaging system (Roper Scientific, Trenton, NJ, USA). Luminescence intensity was quantified using a microplate luminescence reader (Varioskan Flash; Thermo Scientific, Waltham, MA, USA).

### 4.5. Transient Expression

The *ripP1* gene was amplified from the BD-RipP1 plasmid and cloned into the binary expression vector pGDGm at the *Xho*I/*Hind*III restriction sites to generate pGDGm:RipP1. Then, the empty pGDGm vector and the recombinant pGDGm:RipP1 plasmid were separately transformed into *A. tumefaciens* GV3101 competent cells by electroporation (Bio-Rad, Hercules, CA, USA). For *Agrobacterium*-mediated infiltration, GV3101 cultures harboring the relevant binary plasmids were adjusted to an OD_600_ of 0.3, 0.03, or 0.003. These cultures were then infiltrated into the abaxial epidermis of fully expanded leaves from four-week-old *N. benthamiana* plants using a syringe.

### 4.6. Trypan Blue Staining and 3, 3′-Diaminobenzidine (DAB) Staining

For trypan blue staining, agroinfiltrated *N. benthamiana* leaves were collected and boiled in a 1:1 mixture of 96% ethanol and staining solution (10 mL lactic acid, 10 mL phenol, 10 mL glycerol, 10 mL H_2_O, and 10 mg trypan blue) for approximately 5 min until its green color had completely disappeared. The leaves were thereafter destained in destaining solution (dissolve 250 g chloral hydrate into 100 mL H_2_O) overnight. For DAB staining, agroinfiltrated *N. benthamiana* leaves were collected and immersed in 1 mg/mL DAB-HCl (pH 3.8). After vacuum infiltration for 5 min, the leaves were placed on a standard laboratory shaker and shaken at 80–100 rpm for 8 h. All staining procedures were performed in the dark. Afterwards, the leaves were destained in a bleaching solution (ethanol:acetic acid:glycerol = 3:1:1) for 15 min.

### 4.7. Subcellular Localization in N. benthamiana Leaves

The *FRL4a* gene was amplified from the AD-FRL4a plasmid and cloned into the binary expression vector pGDmcherry at *Eco*RI/*Sal*I restriction sites to generate mcherry-FRL4a. Consequently, the plasmids pGDGm:RipI, mcherry-FRL4a, and pGDmcherry were separately transformed into *A. tumefaciens* GV3101 competent cells by electroporation. For *Agrobacterium*-infiltration, GV3101 cultures harboring the pGDGm:RipI and mcherry-FRL4a or pGDGm:RipI and pGDmcherry plasmids were adjusted to an OD_600_ of 0.3. These cultures were then infiltrated into the abaxial epidermis of fully expanded leaves from four-week-old *N. benthamiana* plants using a syringe. At 36 hpi, fluorescence images were captured using a confocal laser-scanning microscope (Leica Model TCS SP8; Leica, Wetzlar, Germany). GFP and mCherry fluorescence signals were detected at excitation wavelengths of 514 nm and 580 nm, respectively.

### 4.8. VIGS Assay in N. benthamiana

The cDNA fragments of 348 bp and 358 bp, corresponding to *FRL4a* and *gfp*, were amplified from the AD-FRL4a and pGDGm constructs, respectively, and inserted between *Eco*RI and *Bam*HI restriction sites of TRV-RNA2 (pTRV2) to generate pTRV2-FRL4a and pTRV2-GFP. For the VIGS assay, pTRV1 or pTRV2 derivatives were individually introduced into *A. tumefaciens* GV3101 competent cells by electroporation. *A. tumefaciens* cultures containing pTRV1 or pTRV2 derivatives were adjusted to an OD_600_ of 0.6, mixed at 1:1 ratio, and incubated with shaking at 26 °C for 3 h. The mixture cultures were then infiltrated into the abaxial epidermis of the lower leaf of 4-leaf stage *N. benthamiana* plants using a syringe. At 10–14 dpi, the newly developed upper leaves were collected and used for subsequent plant assays.

### 4.9. Generation of FRL4a-Overexpressing Transgenic N. benthamiana

To generate *FRL4a*-overexpressing plants, the *FRL4a* gene was cloned from the AD-FRL4a plasmid into the binary vector pCAMBIA1300s at the *Kpn*I/*Bam*HI restriction sites, under the control of the CaMV 35S promoter. The recombinant construct was verified by Sanger sequencing and then introduced into *A. tumefaciens* strain EHA105 prior to plant transformation via the leaf-disc method. All plant transformation procedures were performed by Wuhan BioRun Biosciences Co., Ltd. (Wuhan, China). Transgenic plants overexpressing *FRL4a* were selected using 25 μg·mL^−1^ hygromycin. The primer pair HYG-F (5′-CTGCCCGCTGTTCTACAACCGG-3′) and HYG-R (5′-GGAGCATATACGCCCGGAGTC-3′) was used for PCR amplification to verify transgene integration in T0 and T1 progeny. Homozygous T2 transgenic lines were used for gene expression analysis and disease resistance assays.

### 4.10. Pathogenicity Assays on the N. benthamiana Plants

Pathogenicity assays were conducted on *N. benthamiana* by the root inoculation method as described previously [56]. A cell suspension of *R. solanacearum* was adjusted to a concentration of 1 × 10^8^ CFU/mL and applied to the roots of *N. benthamiana* plants via soil drenching. The severity of wilting symptoms was evaluated daily using the following disease index: 0, no wilting; 1, 1–33% wilted leaves; 2, 34–66% wilted leaves; 3, 67–99% wilted leaves; 4, completely wilted [54]. Disease progression was recorded daily over two weeks post-inoculation. This experiment was performed in triplicate for statistical analysis, with at least six plants used per bacterial strain per replicate.

### 4.11. qRT-PCR Assay

*N. benthamiana* leaves were collected and ground in liquid nitrogen. Total RNA was extracted using the RNAprep Pure Plant Kit (Tiangen Biotech, Beijing, China). The concentration of total RNA was quantified by measuring the OD_260_/OD_280_ ratio, and RNA quality was evaluated via gel electrophoresis. A total of 1 μg of RNA was used for reverse transcription using a reverse transcription kit (GenSTAR, Beijing, China). All primers used for quantitative real-time PCR (qRT-PCR) are listed in Table S2. qRT-PCR assays were performed on a Bio-Rad CFX Connect™ Real-Time System (BIO-RAD, CA, USA) using SYBR Green PCR Master Mix (GenSTAR, Beijing, China). The PCR thermal cycling conditions were as follows: initial denaturation at 95 °C for 30 s, followed by 40 cycles of denaturation at 95 °C for 5 s, annealing at 55 °C for 30 s, and extension at 72 °C for 10 s. The expression of EF1α was used as the internal control.

### 4.12. Western Blotting

Agroinfiltrated leaves (100 mg) expressing RipP1-GFP or FRL4a-mCherry were collected into 1.5 mL EP tubes, and 200 μL of 5 × SDS loading buffer (250 mM Tris-HCl, pH 6.8; 10% SDS; 0.5% bromophenol blue; 50% glycerol; and 5% 2-mercaptoethanol) was added to each tube. The samples were boiled at 100 °C for 5 min, immediately incubated on ice for 2 min, and then loaded onto a 12% SDS-PAGE gel for protein separation. Following electrophoresis, the separated proteins were transferred onto a NC membrane and probed with a primary anti-GFP antibody (1:5000 dilution; TransGen Biotech, Beijing, China) or an anti-mCherry antibody (1:3000 dilution; Abmart, Shanghai, China), followed by a secondary anti-mouse HRP-conjugated antibody (1:3000 dilution; Bio-Rad, USA). Protein bands were visualized using ECL Western blotting detection reagents (GE Healthcare Bio-SciencesGE, Uppsala, Sweden) in accordance with the manufacturer’s instructions.

### 4.13. Sequence Analysis

Sequence alignments were generated using DNAMAN software Version 8.0. A phylogenetic tree was constructed from amino acid sequences utilizing MEGA 12. The evolutionary history of the analyzed taxa was inferred from a bootstrap consensus tree generated with 1000 replicates. The conserved amino acid domains of FRL4a were generated using the Conserved Domain Database (https://www.ncbi.nlm.nih.gov/cdd/).

## Data Availability

The original contributions presented in this study are included in the article. Further inquiries can be directed to the corresponding authors.

## Supplementary Materials

The following supporting information can be downloaded at: https://www.mdpi.com/article/10.3390/plants15071039/s1, Figure S1: Mutation of cysteine 229 in RipP1 does not abrogate its interaction with FRL4a; Figure S2: Fusion of GFP to the C-terminus of RipP1 did not compromise its ability to trigger HR in *N. benthamiana*; Figure S3: RipP1 induces the SA signaling pathway and suppresses the JA and ET signaling pathways in *N. benthamiana*; Figure S4: qPCR analysis validates of *FRL4a* transcript levels in TRV-mediated *FRL4a*-silenced and *FRL4a*-overexpressing transgenic *N. benthamiana* plants; Figure S5: Conserved amino acid sequence alignment of NbFRL4a and its homologous proteins from Solanaceae species; Figure S6: Mosaic leaf phenotype in young leaves of *FRL4a*-silenced *N. benthamiana* plants; Figure S7: NbFRL4a interacts with NbP58^IPK^ in yeast; Table S1: Bacterial strains used in this study [57,58,59]; Table S2: Primers used for RT-qPCR analysis of gene expression in *Nicotiana benthamiana* in this study.
