# Supplementary material for: The Ralstonia solanacearum Effector RipP1 Interacts with Nicotiana benthamiana FRL4a to Suppress Ethylene Signaling and Modulate Bacterial Wilt Susceptibility
Source: Plants (Basel). 2026 Mar 27;15(7):1039. doi: 10.3390/plants15071039 (PMC13075006; doi:10.3390/plants15071039)
Supplement: Supplementary file 1 [file plants-15-01039-s001.zip › plants-4185533-supplementary.pdf]

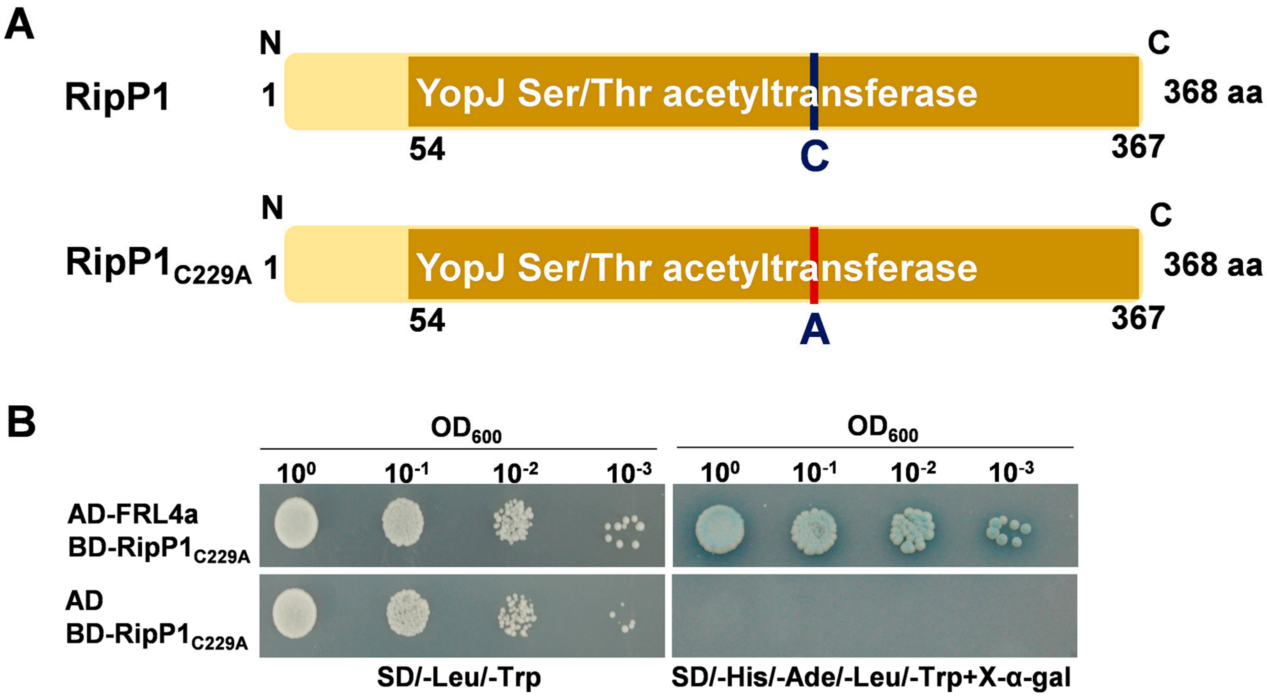

Figure S1. Mutation of cysteine 229 in RipP1 does not abrogate its interaction with FRL4a. **(A)** Schematic diagram of the domain architecture of RipP1 and its catalytic mutant $RipP1_{C229A}$. Amino acid residue positions are indicated by numbers. The YopJ Ser/Thr acetyltransferase domain is shown as a deep yellow box. The catalytic cysteine (C) residue in the YopJ domain of RipP1 is marked with a blue vertical tick; the alanine (A) residue replacing C229 in $RipP1_{C229A}$ is marked with a red vertical tick. **(B)** Y2H assay demonstrated that $RipP1_{C229A}$ retained the ability to interact with FRL4a. Transformant preparation, serial dilution, spotting and incubation conditions were identical to those described in Figure 1A.

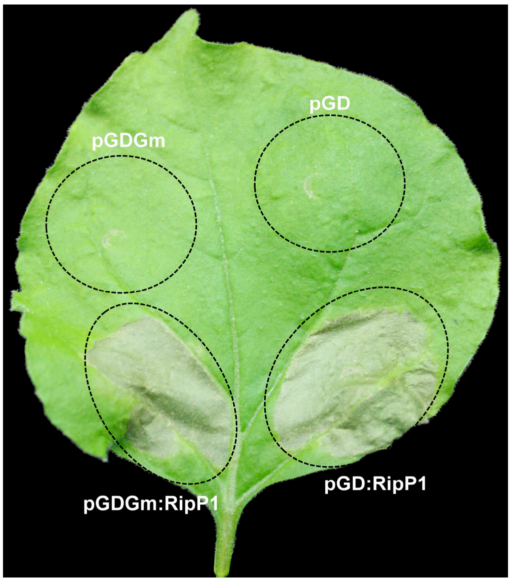

Figure S2. Fusion of GFP to the C-terminus of RipP1 did not compromise its ability to trigger HR in *N. benthamiana*. *A. tumefaciens* GV3101 harboring pGDGm:RipP1 (expressing C-terminal GFP-tagged RipP1) or pGDRipP1 (expressing RipP1 with no tag) was adjusted to OD_{600}=0.3, respectively; GV3101 harboring the empty vectors pGDGm or pGD was adjusted to OD_{600}=0.3, respectively. Photographs were taken at 3 dpi.

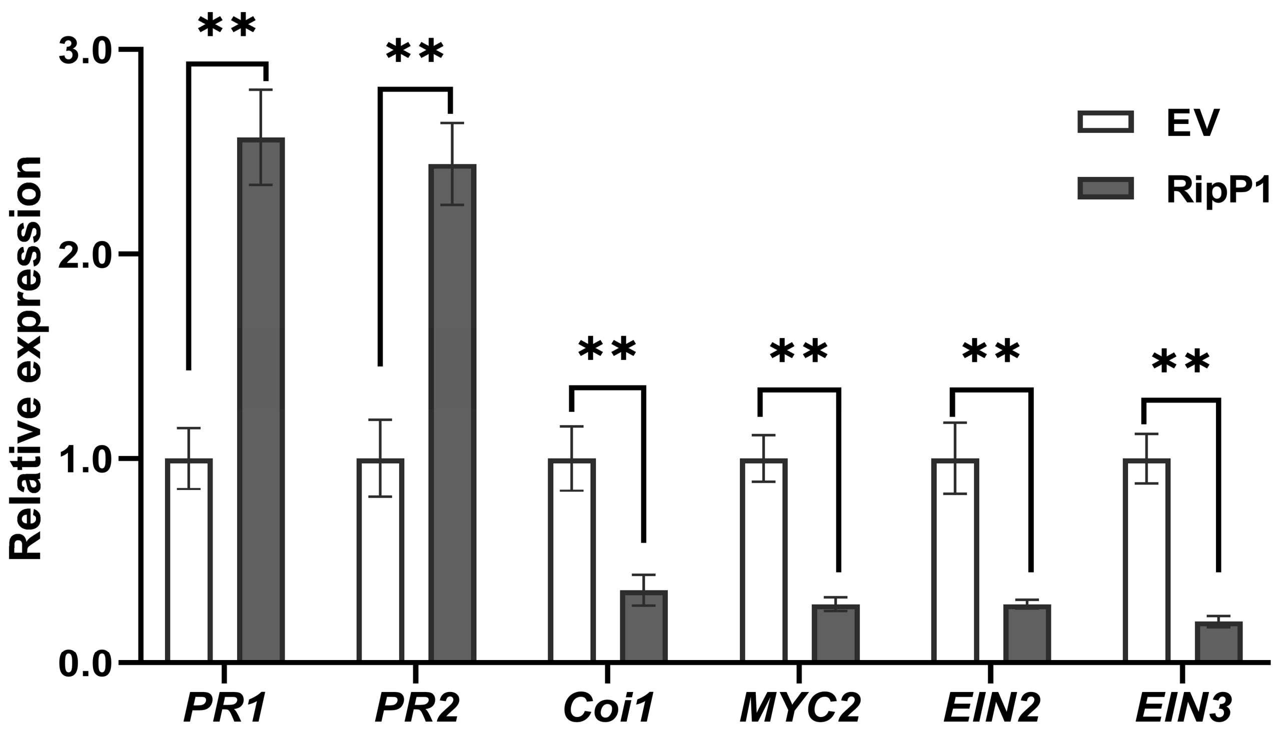

Figure S3. RipP1 induces the SA signaling pathway and suppresses the JA and ET signaling pathways in *N. benthamiana*. Light columns: Leaves were agroinfiltrated with GV3101 harboring pGDGm (empty vector); dark columns: Leaves were agroinfiltrated with GV3101 harboring pGDGm:RipP1. *N. benthamiana* leaves were infiltrated with the above strains at an OD_{600} of 0.03. Total RNA was isolated at 24 hpi. Error bars represent SD from three independent experiments. Statistical significance was determined by Student's *t*-test (**$P < 0.001$, *$P < 0.05$, ns = not significant, $P \geq 0.05$).

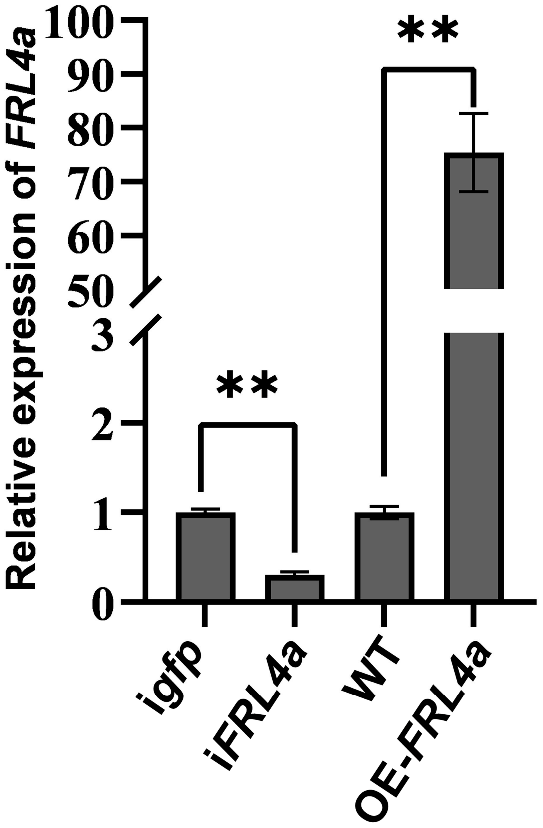

Figure S4. qPCR analysis validation of *FRL4a* transcript levels in TRV-mediated *FRL4a*-silenced and *FRL4a*-overexpressing transgenic *N. benthamiana* plants. Error bars represent SD from three independent experiments. Statistical significance was determined by Student's *t*-test (**P < 0.001).

```
NbFRL4a    SDKSLAEVLRIYCRRMDASGLVTFIQTKRKESTGLRTEIV AALDTCVDPMRLILDAAEEFVGMKVEVKMMGADRRWAYDM    80
NtFRL4a    SEKSLAEVLRIYCRRMDASGLVRFLQTKRKESTGLRTEIV AALDTSVDPMRLILDAAEEFVGMKVEGKMIGADRRWACDM    80
SlFRL4a    SEKSLAEVLRSYCKRMDASGLVGFIQSKRKEPAGLRMEIA AALESSVDPMRLILDAAEEFVGRKVEKKAILADRRWACDM    80
StFRL4a    SEKSLAEVLRSYCKRMDASGLVGFIQTKRKEPTGLRMEIA AALESSVDPMRLILDAAEEFVGRKVEKKAILADRRWACDM    80
CaFRL4a    SDKSLEEVLGVYCRRMDASGLVRFIQTKRKESIGLRAEIG AALQCSLDQMRLILDAAEEFVGMKVEGKTVLSDRRWAYDM    80
LfFRL4a    ...SLSEMLRIYCTRMDASGLVRFLHTKRKESAVLRTEIA PALHSSVDPMRLILDAAEEFVAMKVEGKIRMAERRWACDT    77
Consensus     sl e l  yc rmdasglv f   krke   lr ei  al    d mrlildaaeefv  kve k     rrwa d

NbFRL4a    LVQSVGPVVEGGYGAGRSLKERAARVLEKWRGVMGSGERT SGVCAAEATMFLQLVISFALKERFEEEFLRKLVMEFANRK   160
NtFRL4a    LVQSVGPVVEGGYGAGRSLKERAARVLEKWKGVLGSGDRT SGVCAAEATMFLQLVISFALKERFEEEFLRKLVMEFANRK   160
SlFRL4a    LIQSVVPVVEGGYGAGRSLKERAARVLEKWKGVLGGGDRN SGVCAAEATMFLQLVIAFALKERFEEKFLRKLVLEFANRK   160
StFRL4a    LIQSVVPAAEGGYGAGRSLKERAARVLEKWKGVLGGGDRS SGVCAAEATMFLQLVIAFALKERFEEEFLRKLVLEFANRK   160
CaFRL4a    LIQSVVPSAEGGYGAGRSLKERAARVLEKWKGVLGGGDRG SGVCAAEATMFLQLVITFGLKERFEEEFLRKLILEFANRK   160
LfFRL4a    LFQSVVPVVEGDYGAGRSLKERAARVLEKWKGVLGGGDRN SGVGAAEATMFLQLVIAFALRERFEEGFLRKLVMEFASRK   157
Consensus  l qsv p   eg ygagrslkeraarvlekw gv g g r  sgv aaeatmflqlvi f l erfee flrkl  efa rk

NbFRL4a    DMPKLTVA.FGFGNKIGDIIEELVKSGKEVEAVYFAYESGLAERFPIVSLLKRCLRTCRRNSSNISKEGRFSSAAVDKAN   239
NtFRL4a    DMPKLAVA.FGFGNKIVDIIEELVKSGKEVEAVYFAYESGLAERFPPVSLLKAYLRNCRRNSNNISKKGRFSSAAVDKAN   239
SlFRL4a    DMPKLAVA.FGFGNKIGDIIEELLKSGKEVEAVYFATESGLSERYPPLSLLKLSLRNCRRNANNISKKGKFSPAAVEKAN   239
StFRL4a    DMPKLAVA.FGFGNKIGDIIEELLKSGKEVEAVYFATESGLSERYPPLSLLKLSLRNCRRNANNISKKGKFSSAAVDKAN   239
CaFRL4a    DMPKLAVVTFGSGNKIVDIIEELVKTDKDVEAVYFASEAGLTERYPPLSLLRSALRSCRRNANNISKKGKFSSSAVDKAN   240
LfFRL4a    DMPKLAVA.LGFGNKIADIIEELVKSGKEIEAVYFASESGLAEQFPPLSLLKAYLRNCRRNANNISRKAKFNSAAVERAN   236
Consensus  dmpkl v   g gnki diieel k  k  eavyfa e gl e  p  sll   lr crrn  nis     f   av  an

NbFRL4a    IIELEATKAIIKCVEDHKLEQEFSLEGLKKRVTELEESRAKRKKGT                                   285
NtFRL4a    IIEWEATKAIIKCVEDHKLEQEFSLEGLKKRVTELEQSRAKRKKGT                                   285
SlFRL4a    SIELEATKALIKCVEDHKLEQEFTLEGLKQRVTELEQAKAKKKIGT                                   285
StFRL4a    SIELEATKALIKCVEDHKLEQEFTLEGLKQRVTELEQAKAKKKMGT                                   285
CaFRL4a    TIELEATKALIKCVEDHKLEEEFSLNGLKKRVTELELAKAKRKKST                                   286
LfFRL4a    SSELEATKAIIKCVEDHKLEAEFSLEGLRTRVTELEQAKAEKKKGT                                   282
Consensus    e eatka ikcvedhkle ef l gl  rvtele   a  k  t
```

Figure S5. Conserved amino acid sequence alignment of NbFRL4a and its homologous proteins from Solanaceae species.

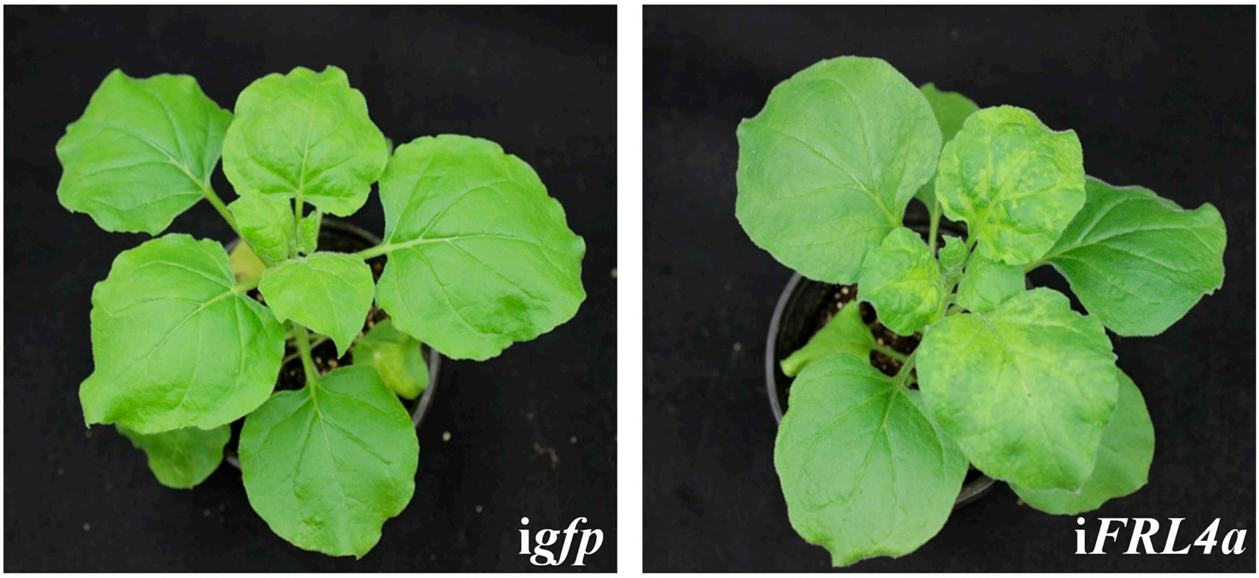

Figure S6. Mosaic leaf phenotype in young leaves of *FRL4a*-silenced *N. benthamiana* plants. Left: control plants (i*gfp*); Right: i*FRL4a* silenced plants.

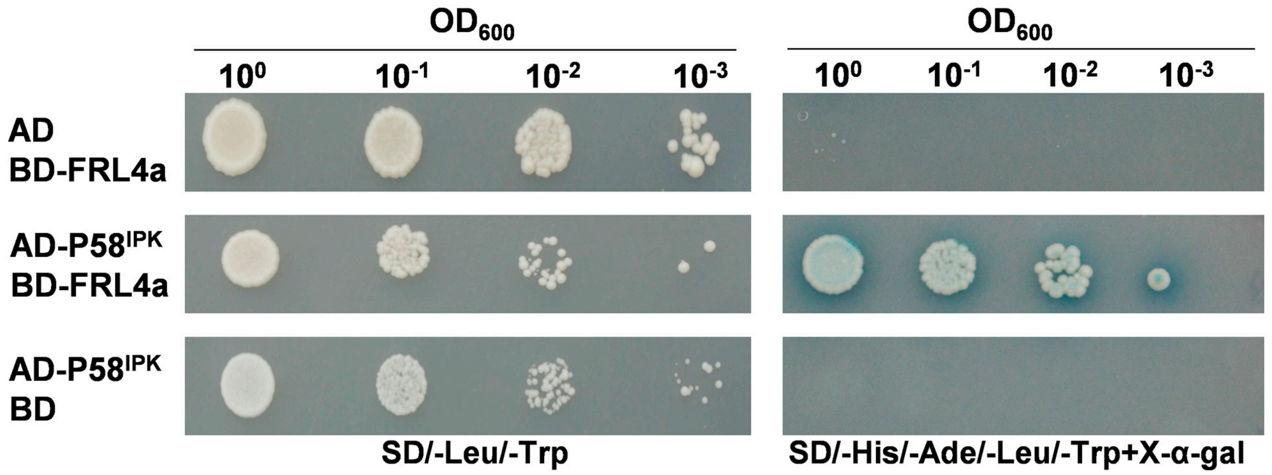

Figure S7. NbFRL4a interacts with NbP58^{IPK} in yeast. Transformant preparation, serial dilution, spotting and incubation conditions were identical to those described in Figure 1A. The yeast strain AH109 harboring plasmid combinations AD/BD-FRL4a or AD-P58^{IPK}/BD served as negative controls.

Table S1. Bacterial strains used in this study

Strains	Relevant characteristics	Resource/Reference
Escherichia coli		
DH5α	*F′Φ80dlacZDM15D(lacZYA-argF)U169 endA1 deoR recA1 hsdR17(rK2 mK+) phoA supE44 l2 thi-l gyrA96 relA1*	Clontech
BL21(DE3)	*F-, ompT, hsdSB (rB-mB-), gal, dcm*	Novagen
Ralstonia solanacearum		
GMI1000	PB^r, wild-type *R. solanacearum*, phylotype I, biovar 3, race 1	[57][1]
FJ1003	PB^r, wild-type *R. solanacearum*, phylotype I	[58][1]
Agrobacterium tumefaciens		
GV3101	Rif^r, with Ti plasmid pMP90	[59][1]

[1] The references appear in the text’s reference list.

Table S2. Primers used for RT-qPCR analysis of gene expression in *Nicotiana benthamiana* in this study

Primer pair	Sequence (5'-3')	Length of target fragment (bp)
NbEF1α-F	TGGTGTCCTCAAGCCTGGTATGGTTG	155
NbEF1α-R	CGCTTGAGATCCTTAACCGCAACATTCTT	
NbFRL4a-F	GGAATCCTCCTCCATCACATCA	92
NbFRL4a-R	GACCGTGCTAGGTGCCTCGTAT	
Nbhin1-F	CTGCAACCCATGTAGCTGTCTC	124
Nbhin1-R	TTTGTTAGGACGAAGAACGAGCC	
NbPR1-F	ATGGTCAATACGGCGAAAAC	188
NbPR1-R	CCTAGCACATCCAACACGAA	
NbPR2-F	CAGCGGCAGGGTTGCAAGAT	112
NbPR2-R	AAACTCTTGAATTCTTCGCG	
NbCoi1-F	GAGAAGAGAGAATAACAGATC	124
NbCoi1-R	CTGAGACCTACATCAGTAAG	
NbMYC2-F	GAAAAGAGGCCAAAGAAGCGAGGAA	198
NbMYC2-R	CTTCAGCTCATTAATATATGAAATT	
NbEIN2-F	ACACCAGAAGCGAAGTCCAAGA	139
NbEIN2-R	CACTCCCAGATGGAATATACGC	
NbEIN3-F	TTAGGGAATGGTGGAAAG	136
NbEIN3-R	TGCAAGGTATGAGGCGTA	
